# The Complex Network of ADP-Ribosylation and DNA Repair: Emerging Insights and Implications for Cancer Therapy

**DOI:** 10.3390/ijms241915028

**Published:** 2023-10-09

**Authors:** Ziyuan Li, Aiqin Luo, Bingteng Xie

**Affiliations:** Key Laboratory of Molecular Medicine and Biological Diagnosis and Treatment (Ministry of Industry and Information Technology), School of Life Science, Beijing Institute of Technology, Beijing 100081, China

**Keywords:** DNA damage and repair, post-translational modification, ADP-ribosylation, PARP inhibitors, cancer therapy

## Abstract

ADP-ribosylation is a post-translational modification of proteins that plays a key role in various cellular processes, including DNA repair. Recently, significant progress has been made in understanding the mechanism and function of ADP-ribosylation in DNA repair. ADP-ribosylation can regulate the recruitment and activity of DNA repair proteins by facilitating protein–protein interactions and regulating protein conformations. Moreover, ADP-ribosylation can influence additional post-translational modifications (PTMs) of proteins involved in DNA repair, such as ubiquitination, methylation, acetylation, phosphorylation, and SUMOylation. The interaction between ADP-ribosylation and these additional PTMs can fine-tune the activity of DNA repair proteins and ensure the proper execution of the DNA repair process. In addition, PARP inhibitors have been developed as a promising cancer therapeutic strategy by exploiting the dependence of certain cancer types on the PARP-mediated DNA repair pathway. In this paper, we review the progress of ADP-ribosylation in DNA repair, discuss the crosstalk of ADP-ribosylation with additional PTMs in DNA repair, and summarize the progress of PARP inhibitors in cancer therapy.

## 1. Overview of ADP-Ribosylation and Its Importance in DNA Repair

Protein adenosine diphosphate (ADP)-ribosylation was first proposed in the early 1960s [1]. It involves the transfer of ADP-ribose (ADPr) from nicotinamide adenine nucleotide (NAD) to the target protein and the release of nicotinamide (NAM). This modification includes both mono-ADP-ribosylation (MAR) and poly-ADP-ribosylation (PAR). ADP-ribosylation plays a critical role in numerous biological processes such as DNA damage repair, gene regulation, and energy metabolism [2,3,4,5,6,7,8,9]. ADP-ribosylation is primarily catalyzed by ADP-ribosyltransferases (ARTs) [10], which is ubiquitous in cells and is associated with a growing number of biological processes, including DNA repair, replication, transcriptional regulation, intracellular and extracellular signaling, viral infection, cell death, and progression of mitosis.

The most well-known function of ADP-ribosylation is in DNA damage repair, where ADP-ribosylation works in a variety of ways. On the one hand, ADP-ribosylation can recruit proteins involved in DNA damage repair by interacting with the BRCT domains of these proteins. Previous studies have demonstrated that ADP-ribosylation occurs following the interaction of poly(ADP)-ribosylase 1 (PARP1) with the DSB terminus, leading to the recruitment and involvement of the DNA ligase XRCC1 and additional related repair proteins at the DNA damage site for efficient DNA repair [11]. On the other hand, ADP-ribosylation can also affect DNA repair through crosstalk with different post-translational modifications, including ubiquitination, methylation, acetylation, phosphorylation, and SUMOylation. These protein post-translational modifications interact with ADP-ribosylation in distinct ways that affect the DNA damage repair pathway.

PARP has been recognized as an effective target for anticancer therapy to achieve cell death induced by DNA damage. Numerous studies have highlighted the significant efficacy of PARP inhibitors in breast, ovarian, and prostate cancers with BRCA1/2 mutations. The presence of mutations in the BRCA1/2 gene results in homologous recombination (HR) deficiency, necessitating the reliance of cancer cells on alternative DNA repair pathways to compensate for this deficiency. Thus, HR-deficient cancer cells exhibit superior susceptibility to PARP1 inhibitors due to their involvement in non-HR repair mechanisms. Although PARP inhibitors have been widely used in cancer therapy, many patients will develop resistance toward these inhibitors or relapse. As research has deepened, numerous methods have been developed in recent years to address the resistance of PARP inhibitors.

This review aims to summarize the recent developments of ADP-ribosylation in DNA repair, focusing on the crosstalk of ADP-ribosylation with additional protein post-translational modifications and the progress of PARP inhibitors in cancer therapy.

### Catalog

1. Overview of ADP-ribosylation and its importance in DNA Repair
2. Key proteins of ADP-ribosylation in DNA repair
  (a) Writers;
  (b) Erasers;
  (c) Cofactors.
3. Roles of ADP-ribosylation in DNA damage repair
  (a) Recruitment of DNA repair factors;
  (b) Novel roles of ADP-ribosylated proteins.
4. Crosstalk of ADP-ribosylation with other protein post-translational modifications
  (a) Ubiquitination;
  (b) Methylation;
  (c) Acetylation;
  (d) Phosphorylation;
  (e) SUMOylation.
5. Research progress of PARPi
  (a) PARPi-related cancers and their drugs;
  (b) Mechanisms of action of PARPi;
  (c) Mechanisms of drug resistance to PARPi;
  (d) Next generation PARPi;
  (e) Advancements in PARPi resistance solutions;
6. Conclusions and future prospects.

## 2. Key Proteins of ADP-Ribosylation in DNA Repair

**Figure 1:**
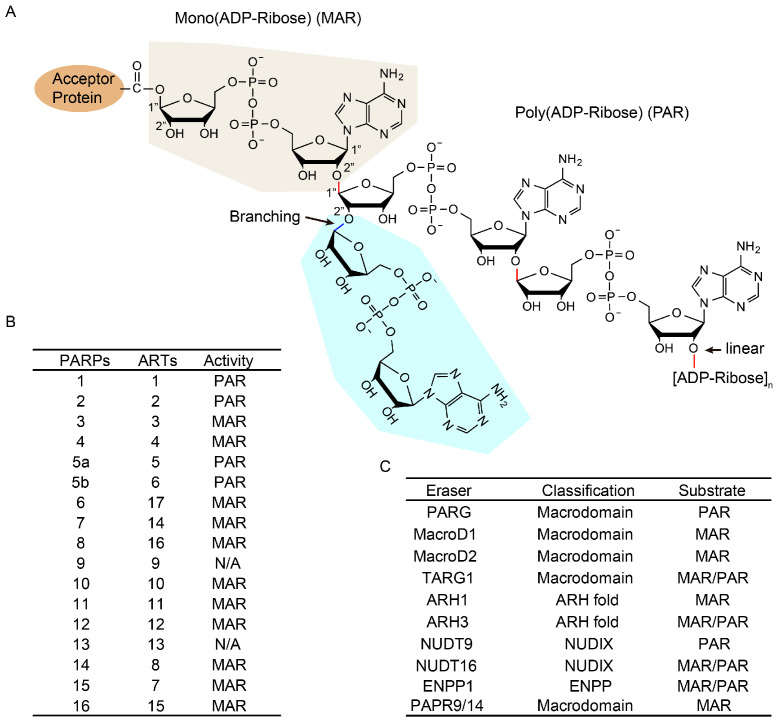
Overview of ADP-ribosylation and its writers and erasers: (**A**) schematic representation of the structure of poly(ADP)-ribosylation; (**B**) known ADP-ribosylation writers [16] and (**C**) erasers and their catalytic properties (PARG [24], MacroD1 [25], MacroD2 [28], TARG1 [26], ARH1 [29], ARH3 [27], NUDT9 [31], NUDT16 [32], ENPP1 [33], PARP9/14 [34]).

ADP-ribosylation is a reversible post-translational modification (PTM), and the completion of the ADP-ribosylation cycle cannot be achieved without the help of writers and erasers, as well as essential cofactors (Figure 1). The main function of the ADP-ribosylation writers is to split NAD into NAM and ADPr, and then transfer ADPr to various targets in turn. The main writer of ADP-ribosylation is ADP-ribosyltransferase. ARTs transfer one or more ADP-ribose units of NAD to targeted proteins on a variety of amino acids, including serine (Ser) [12], threonine (Thr) [13], lysine (Lys), arginine (Arg), glutamic acid (Glu), aspartate, (Asp), and cysteine (Cys). The ART family consists of 17 enzymes [14]. PARylation can be catalyzed by PARP1, PARP2, and tankyrase 1/2 [15]. Almost all other family members are MAR-transferases [16]. PARP1, PARP2, and PARP3 belong to the class of DNA-dependent ARTs whose activity is triggered directly by DNA damage through the zinc finger and/or WGR DNA binding domains. They are involved in several DNA repair mechanisms, including base excision repair (BER) and double-strand break repair pathways (DSBR) [17]. PARP1, PARP2, and PARP3 share a conserved C-terminal structure, which includes the Trp–Gly–Arg domain and the catalytic domain (CAT). Additionally, PARP1 has three zinc finger domains at the N-terminus that bind to damaged DNA. It also possesses a self-modifying domain known as the BRCT domain, which functions as a receptor for ADP-ribose fragments and enables PARP1 to mediate protein–protein interactions through its self-PARylation. PARP1 generally binds to DNA damage sites and recruits relevant DNA damage repair factors to promote DNA damage repair and maintain gene stability. A recent study has shown that PARP1 can bind to alternative DNA structures to maintain genomic stability, including stagnant replication forks [18] and R loops [19]. The function of PARP2 overlaps with that of PARP1, and PARP2 is recruited to the site of damage and activated by PARP1, which subsequently catalyzes branching PARylation at the site of damage [20]. PARP3 can accelerate the nonhomologous end junction (NHEJ) response to DSB. Tankyrase 1 and tankyrase 2 are DNA-independent enzymes whose activity is not triggered directly by DNA damage. These two enzymes share most of their protein companions, resulting in overlapping biological functions in WNT/beta-catenin signaling, mitosis, apoptosis, viral replication, and proteasome regulation [21]. It has been shown that tankrase 1 plays a role in the DDR (DNA damage response) by being recruited to focal sites in X-ray cells through MERIT40, a component of the BRCA1-A and BRISC complex [22]. 

ADP-ribosylation is a reversible modification capable of being dynamically erased in the cell. PARG is the main eraser enzyme [23], and the importance of PARG catalytic activity has become apparent. PARG-depleted cells exhibit hypersensitivity to genotoxic damage, indicating that efficient PARG-mediated PAR turnover is essential for DNA damage repair. The presence of PARG prevents the excessive production of PAR during chronic replication stress. However, PARG cannot act on the protein-ribose bond at the end, and its catalytic efficiency is low for short polymers containing less than four units. Additional eraser enzymes include amino acid-specific ADP-ribose receptor hydrolases (e.g., MacroD1 and MacroD2 [24], terminal ADP-ribose protein glycohydrolase 1 (TARG1) [25], ADP-ribose hydrolase (ARH) family members ARH1 and ARH3 [26], and several phosphodiesterases). MacroD1, MacroD2, and TARG1 can destroy the O-glycosidic bonds of modified aspartate glutamate and O-acetyl-ADPR [27]. MacroD1, MacroD2, and TARG1 can reverse any MARylation produced by the PARP enzyme and also remove the last ADP-ribose left by PARG. ARH1 can specifically reverse MARylation via arginine [28]. ARH3 specifically clears the Ser-ADPr glycosidic bond and entirely removes the ADPr chain attached to the serine residue in the protein. NUDT9 [29] and NUDT16 [30] are NUDIX hydrolases, which belong to the nucleoside-linked partial X (NUDIX) protein superfamily. They cleave pyrophosphate bonds and produce phosphoribose-AMP of PAR chains or AMP of MARylated proteins as their primary reaction products. Although ENPP1 [31], a pyrophosphatase, does not have a NUDIX domain, it has the ability to digest PAR and MAR modifications similar to the NUDIX enzyme. Recent studies have found that PARP9 and PARP14 exhibit ADP-ribosylated glycohydrolase activities [32]. Macrodomains 1 of PARP14 and PARP9 are capable of cleaving ADP-ribosylated arginine.

PARP cannot fulfill its function without the assistance of numerous cofactors, such as histone PARylation factor 1 (HPF1), phosphatase 1 nuclear-targeting subunit 1 (PNUTS), TSG101, and various other factors involved in ADP-ribosylation in the DNA damage repair response. Previous studies have shown that PARylation primarily occurs on serine residues, and this process is closely related to the HPF1 factor, which changes the amino acid specificity of PARP1/2 from aspartate/glutamate to serine residues [33]. However, the mechanism through which the HPF1 factor influences PARP recruitment to DNA damage remains unclear. As research has progressed, the active sites where HPF1 exerts its function have been discovered. It has been shown that HPF1 regulates the number and length of ADP-ribose chains of histones [34]. TSG101 is also required for PARP1 activation. TSG101 interacts with PARP1 through its own coil domain to stimulate the PARylation of cells [35]. The depletion of TSG101 completely eliminates cell PARylation and results in PARP1 capture in the DNA lesion due to the loss of its own PARylation. Phosphatase 1 nuclear-targeting subunit 1 (PNUTS) is a powerful cofactor in PARP1’s recruitment to DNA damage sites. PNUTS bind to the BRCT domain of PARP1 and mediate PARP1 recruitment to DNA damage sites [36]. In addition to the factors described above, an increasing number of cofactors are emerging.

## 3. Roles of ADP-Ribosylation in DNA Damage Repair

### 3.1. Recruitment of DNA Repair Factors

ADP-ribosylation plays an essential role in DNA damage repair, primarily through the involvement of PARP. The presence of the PAR strand increases the amount of negative charge in the DNA lesion, thereby relaxing the chromosome structure through electrostatic repulsion between negatively charged DNA and PAR. In addition, PARs are recognized by PAR-binding modules found in numerous chromatin remodeling complexes and DNA damage repair factors. These modules mediate the recruitment of DNA damage repair machinery to the sites of DNA damage, promoting chromosome remodeling and facilitating DNA damage repair. For example, PARP1 and PARP2 are activated when they bind to SSB. By promoting the recruitment of XRCC1 and ALC1 to the damage site via the ADPr of the target protein at the break, it modulates the assembly and turnover of additional factors that promote DNA repair [37].

As the first member of the PARP family, PARP1 plays an influential role in the recruitment of DNA damage factors. PARP1 consists of six domains [38], four of which are related to DNA lesions, including three zinc finger mods in the N-terminus and one WGR domain connected to the zinc finger through the BRCT domain. PARP1 is initially recruited to the DNA damage site by its N-terminus, while its C-terminus stimulates ribosylation. PARP1-induced ADP-ribosylation at the DNA damage site is a prerequisite for the recruitment of additional DNA repair factors and enzymes at the DNA damage site. It has been shown that the recruitment of PARP2 to DNA damage sites must be based on PARP1 activation. The recruitment times of PARP1 and PARP2 to the DNA damage sites are different. When DNA damage occurs, PARP1 is immediately recruited to the DNA damage region, leading to a significant increase in ADP-ribosylation. This ADP-ribosylation then triggers the accumulation of PARP2 at the damaged DNA site [20]. PARP1 and PARP2 are activated after recruitment to the DNA damage site to generate PAR and MAR chains. DNA damage repair factors recognize PAR or MAR strands and act at the site of DNA damage.

### 3.2. Novel Roles of ADP-Ribosylated Proteins

PARP1 enables the ADP-ribosylation of histone and chromatin remodeling-related proteins. The glutamate residue 141 (E141) of H2AX, a histone H2A variant, was identified as an ADP-ribosylation site. E141 ADP-ribosylation helps recruit Neurex3 glycosylase to DNA damage sites to remove damaged bases during base excision repair following oxidative DNA damage [39]. MORC2 is a chromatin-remodeling enzyme. After DNA damage, PARP1 interacts with MORC2 to recruit it to the site of DNA damage. PARylation stimulates the activity of MORC2 ATPase to promote chromatin remodeling and DNA repair [40]. ADP-ribosylation also has a negative regulatory effect on DNA damage repair. Ataxic telangiectasia mutation (ATM) is a major regulator of the DNA damage response in eukaryotes. Studies have shown a significant increase in PARylation in ATM-deficient cells. The surge in the PARylation of PARP causes the disordered proteins that would otherwise be scattered to aggregate, thus affecting the repair of DNA damage [41]. ADP-ribosylation has long been known to be a protein-specific modification, and recent studies have shown that nucleic acids are also targets of ADP-ribosylation [27,42,43], which is regulated by the DarT-DarG toxin–antitoxin system, unlike the traditional ADP-ribosylation on proteins [44].

## 4. Crosstalk of ADP-Ribosylation with Other Protein Post-Translational Modifications

**Figure 2:**
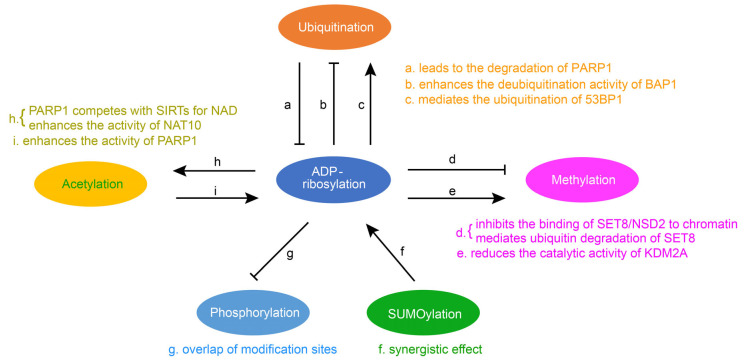
Crosstalk of ADP-ribosylation with other protein post-translational modifications. Summary of interaction relationships for ADP-ribosylation and the remaining five modifications, i.e., ubiquitination, methylation, acetylation, phosphorylation, and SUMOylation. Examples are given to illustrate the relationship between them (a [52], b [53], c [54], d [55,56], e [57], f [62], g [61], h [58,59], i [60]).

Post-translational modification is central to regulating protein activity, stability, subcellular localization, and partner interaction. They considerably extend the functionality and diversity of the proteome and have become key players in the regulation of numerous cellular and physiological processes. As research has deepened, in addition to a single regulatory PTM, numerous proteins have been modified by multiple different types of PTM in a coordinated fashion to regulate biological outcomes. A pathway can be affected by multiple PTMs. The interaction between two PTMs involves two cases. One is positive crosstalk, where the first PTM promotes the formation or function of the second PTM. The other is negative crosstalk, where the first PTM hinders the formation or function of the second PTM. ADP-ribosylation can be in crosstalk with other modifications, such as ubiquitination, methylation, acetylation, phosphorylation, and SUMOylation (Figure 2).

### 4.1. The Crosstalk between Ubiquitination and ADP-Ribosylation

Protein ubiquitination is one of the most significant post-translational modifications, and it leads to the degradation of proteins through proteasome or lysosome. The ubiquitin–proteasome system consists of a ubiquitin-activating enzyme (E1), a ubiquitin-coupled enzyme (E2), and a ubiquitin ligase (E3). E1 activates ubiquitin and transfers it to E2, and E3 specifically recruits ubiquitin protein substrates. The crosstalk between ubiquitin and ADP-ribosylation is mainly through E3. It has been reported that PARP1 is the substrate of ubiquitin ligase and that ADP-ribosylation is primarily affected by ubiquitin through the interaction of PARP1 with ubiquitin ligase E3. For example, as a ubiquitin ligase, WWP2 can mediate the polyubiquitination of PARP1 and lead to the degradation of PARP1 [45]. BAG3 is capable of binding to the BRCT domain of PARP1 and enhancing the activity of the E3 ubiquitin ligase WWP2, thereby promoting ubiquitination and subsequent degradation of PARP1 [46]. TRIP12, another ubiquitin ligase E3, binds PARP1 via the central PAR binding WWE domain and catalyzes PARP1 polyubiquitination by its carboxy-terminal HECT domain, triggering proteasome degradation and preventing PARP1 accumulation [47]. ADP-ribosylation can also affect deubiquitination. BAP1, a ubiquitin C-terminal hydrolase domain, can promote the repair of DNA damage induced by ultraviolet light (UV) through deubiquitination activity. PARP1 is able to recruit BAP1 to the site of damage and enhance the deubiquitination activity of BAP1 through PARylation [48]. ADP-ribosylated 53BP1 mediates the ubiquitination and degradation of 53BP1 in response to DNA damage. Recent studies have found that a hydrolase, NUDT16, can remove ADP-ribosylation from 53BP1 and inhibit the ubiquitin degradation of 53BP1, which helps to stabilize the 53BP1 protein and enables it to recruit to the histone methylation site for function [49].

### 4.2. Crosstalk between Methylation and ADP-Ribosylation

Histone methylation is an important epigenetic modification that does not alter the charge. The crosstalk between histone methylation and ribosylation significantly impacts cell activity. The presence of ADP-ribosylation could reduce the catalytic activity of methyltransferase or demethyltransferase. SET8 is the only histone methyltransferase that manipulates histone H4K20, and it has been reported that PARP1 can interact with SET8, thereby inhibiting SET8′s binding to DNA and nucleosomes and affecting the monomethylation of histone H4K20. In addition, the binding of SET8 to PARP1 promotes PARylation of SET8 and thus mediates the ubiquitin degradation of SET8 [50]. NSD2 is a histone methyltransferase that functions similarly to SET8. Under oxidative stress, PARP1 can regulate NSD2 through PARylation, which inhibits the binding of NSD2 to chromatin and reduces its recruitment to target genes. This results in the decreased catalytic activity of the NSD2 histone methyltransferase [51]. The crosstalk between methylation and ADP-ribosylation is also reflected in the demethylase KDM2A that can be mono-ADP-ribosylated by the ADP-ribosyltransferase activity of SIRT6, resulting in increased H3K36me2 at the site of DNA damage. Finally, the initiation of transcription is inhibited, and the efficiency of nonhomologous end joining is improved [52].

### 4.3. Crosstalk between Acetylation and ADP-Ribosylation

Acetylation in proteins occurs mainly on lysine residues. Protein acetylation can regulate a variety of protein properties, such as DNA–protein interactions, subcellular localization, transcriptional activity, and protein stability. The crosstalk between acetylation and ADP-ribosylation plays an essential role in various diseases. Sirtuins (SIRTs) are NAD-dependent deacetylases, which play their main functions through deacetylation. PARP1 indirectly regulates the activity of deacetylation and affects related physiological functions. PARP1 competes with SIRTs for NAD, resulting in aging muscle showing clear signs of mitochondrial dysfunction, oxidative stress, and inflammation [53]. ADP-ribosylation can also directly affect the catalytic activity of acetyltransferase. NAT10 is a member of the GNAT family of lysine acetyltransferases, and PARP1 is able to catalyze NAT10 PARylation on three conserved lysines in its C-terminal nucleolus localization signal sequence. It has been shown that the PARylation of the acetyltransferase NAT10 is key to its effects [54]. Additionally, the function of PARP1 can be directly affected by deacetylase. Sirtuin 3 (SIRT3) is a type III histone deacetylase, which can inhibit cardiac hypertrophy. It has been shown that, by interacting with PARP1, SIRT3 inhibits the acetylation of PARP1, thereby reducing the activity of PARP1 to inhibit cardiac hypertrophy [55].

### 4.4. Crosstalk between Phosphorylation and ADP-Ribosylation

Phosphorylation is the first known post-translational modification of a protein. The main players in phosphorylation are protein kinases and protein phosphatases, which phosphorylate and dephosphorylate specific amino acid residues of proteins to regulate the catalytic activity of proteins. It has been shown that the serine ADP-ribosylation and phosphorylation sites greatly overlap [56]. Thus the ADP-ribosylation and phosphorylation crosstalk is mostly negative crosstalk. The presence of ADP-ribosylation suppresses phosphorylation so that phosphorylation-related pathways are weakened or unable to function. It has been shown that PARP1 can perform the ADP-ribosylation of the histone H2B-Glu35 to inhibit the AMP kinase-mediated phosphorylation of the neighboring H2B-Ser36 [57]. ADP-ribosylation at E141 of the histone mutant H2XA inhibits phosphorylation at the adjacent site S139 [58]. PARP1 also inhibits the phosphorylation of STAT3, which can be combined with the promoter of PD-L1 (programmed death ligand 1) to regulate it [59].

### 4.5. Crosstalk between SUMOylation and ADP-Ribosylation

The SUMOylation process is similar to ubiquitination: It consists of maturation, activation, coupling, and defibrination steps. The amino acid sequence of SUMO proteins is similar to that of ubiquitin. The entire process of SUMOylation also requires the involvement of three enzymes, namely the SUMO-activating E1 enzyme, the SUMO-conjugating E2 enzyme, and the SUMO E3 ligase. It has been shown that the SUMOylation of PARP1 abolishes P300-mediated PARP1 acetylation and has no effect on the ADP-ribosylation of PARP1. In addition, SUMOylation inhibits the transcriptional coactivator function of PARP1, resulting in decreased expression of PARP1-regulated genes [60]. TDP1 is tyrosine-DNA phosphodiesterase 1, and it has been shown that the ADP-ribosylation of TDP1 in cooperation with its SUMO can promote protein stabilization and promote its function in repairing the topoisomerase I (TOP1)-trapping cleavage complex [61]. PIASy is a small ubiquitin-associated modifier ligase that mediates the SUMO-2/3 coupling of PARP1 on mitotic chromosomes, and SUMO-2/3 heavily binds to PARP1 on mitotic chromosomes. The polyADP-ribosylase activity of SUMO PARP1 also did not alter the accumulation of PARP1 on mitotic chromosomes. However, SUMO PARP1 has the ability to modify additional chromosomal proteins [62].

## 5. Research Progress of PARPi

### 5.1. PARPi-Related Cancers and Their Drugs

Olaparib, lucaparib, nilaparib, and talazoparib have been approved by the FDA for the treatment of various cancers. According to preclinical studies, different PARP inhibitors have different efficiencies in capturing PARP in a variety of tumor cells, ranging from large to small: talazoparib > nilaparib > lucaparib = olaparib. Based on its revolutionary results in clinical trials, the most successful aspect of PARP inhibitors is that they limit tumor progression without affecting normal cell growth, making them highly suitable treatment options for cancer.

The first PARPi to be validated in clinical trials is olaparib, which has shown promising efficacy and safety in several malignancies, such as ovarian cancer, breast cancer, prostate cancer, pancreatic cancer, etc. A growing number of clinical trials have demonstrated its safety and efficacy. A Phase III trial evaluated the efficacy of olaparib in men with prostate cancer whose disease had progressed during second-generation hormone therapy [63]. Olaparib has been shown to have clinically significant improvements in overall survival compared with placebo [64]. Longer PFS (median progression-free survival) was observed with olaparib in a randomized, double-blind, placebo-controlled, Phase III trial assessing the effect of maintenance olaparib on nonprogressive pancreatic cancer during at least 16 weeks of first-line platinum-based chemotherapy in patients with GBRCA1/2-mutated metastatic pancreatic cancer [65]. Olaparib maintenance therapy significantly increased PFS in patients with metastatic HER2-negative BRCA-mutated breast cancer compared with standard chemotherapy [66]. Lucaparib maintenance therapy is an excellent treatment for patients with platinum-sensitive advanced pancreatic cancer with BRCA1/2 or PALB2 pathogenic variants, primarily in ovarian and prostate cancers. Lucaparib has been shown to be effective in the maintenance therapy of ovarian cancer, particularly in patients with BRAC mutations. A recent single-arm Phase II trial revealed the efficacy and safety of lucaparib in patients with recurrent advanced ovarian cancer. It was approved by the FDA in April 2017. Regardless of HR deficiency, PFS was significantly longer in patients with advanced ovarian cancer treated with nilaparib, which primarily includes ovarian, prostate, breast, and nonsmall cell lung cancers. Based on the results of the PRIMA Phase III randomized trial, mid-term PFS was significantly higher in the nilaparib-treated group than in the placebo group, and an increase in PFS was observed in the nilaparib-treated group regardless of HR deficiency. Talazopanib is approved for the treatment of breast cancer patients with inherited BRCA1/2 mutations due to its efficacy and safety profile [67]. Talazopanib is primarily used to treat ovarian, breast, and prostate cancers. The Phase III Embla study demonstrated a significant PFS improvement with talazopanib monotherapy versus standard chemotherapy in patients with metastatic HER2-negative BRCA-mutated breast cancer [67].

### 5.2. Mechanism of Action of PARPi

PARPi is used in ovarian and breast cancer with mutated breast cancer susceptibility genes (BRCAs) [68]. PARPi and BRAC have a synthetic lethal mechanism of action [69]. PARP inhibitors block the recruitment of the necessary DNA repair pathway mechanism for base-cutting, and defects in BRCA simultaneously prevent homologous recombination, ultimately leading to genomic instability [70,71,72,73,74,75]. PARPi targets cancer cells with DNA repair defects, and the mutated breast cancer susceptibility gene is the most intensively studied. As technology has improved, PARPi has also been shown to work in different cancer cells with DNA repair defects. Several studies have shown that PARPi also has autonomic immunomodulatory properties in ERCC1-deficient cancer cells. With more and more research, PARPi will be found to play an essential role in the treatment of a growing number of cancer types [76,77,78,79,80,81,82,83].

There are two main mechanisms of PARPi action: (1) PARPi can inhibit the catalytic activity of PARP; (2) it can also trap PARP at the site of DNA damage. The persistence of the PARP–DNA complex leads to the arrest of the replication fork, and the collapse of the replication fork generates DSBS. Synthetic lethality refers to the presence of PARPi and defects in HR repair, and the synthetic lethality of PARPi with mutated breast cancer susceptibility genes plays a crucial role in the treatment of related cancers [84,85,86,87,88,89,90,91].

PARP1 is used as an example to make the functionality of PARPi more explicit. When SSB damage occurs in a cell, PARP1 is recruited to the site of SSB damage, and the C-terminal domain of PARP1 is rapidly activated to hydrolyze NAD, resulting in the polyADP-ribosylation of the relevant protein and initiating the repair mechanism. One of the functions of PARPi is that it competes with NAD, and the combination of PARP1 prevents the PAR chain from being generated, causing the SSB to fail repair and leading to DSB generation. The other function is the capture of PARP1 by PARPi, which will inhibit autoparization and prevent PARP1 from being released from DNA. When PARPi binds to PARP1, PARP1 becomes allosteric, thus enhancing the binding to the DNA damage site, resulting in the accumulation of SSB and the generation of DSB that requires HR repair. Without an HR repair system in the cell, it will result in cell death.

### 5.3. Mechanisms of Drug Resistance to PARPi

**Figure 3:**
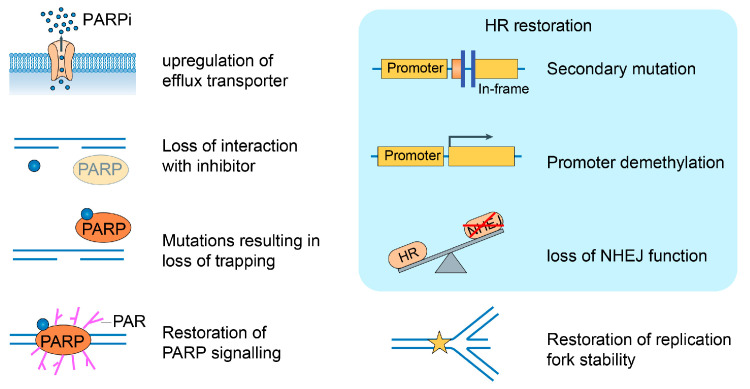
Mechanisms of drug resistance to PARPi. There are numerous reasons for PARPi resistance, including efflux of PARPi, reduced PARP1 trapping, restoration of HR repair, re-establishment of replication bifurcation stability, etc.

With the widespread use of PARPi, a large number of patients have shown resistance to PARPi, which is a great challenge for cancer treatment [92,93,94,95,96,97,98]. There are numerous reasons for resistance, including the efflux of PARPi, reduced PARP1 trapping, the restoration of HR repair, the re-establishment of replication bifurcation stability, etc. (Figure 3). HR repair restoration is one of the main causes of PARPi resistance. HR repair can be reintroduced through BRCA-dependent and BRCA-independent mechanisms and is primarily facilitated by secondary mutations or epigenetic modifications re-establishing the expression of functional proteins involved in HR and the loss of proteins that regulate NHEJ [99]. TP53BP1 is used in combination with the Shieldin complex to promote NHEJ by counteracting DSB terminal resection, resulting in the generation of the DNA substrate needed for HR [100]. The restoration of replication fork stability is also one of the reasons for PARPi. BRAC1 and BRAC2 are essential components to protect the stalled replication fork from nuclease degradation. The ability to use alternative mechanisms that protect against the degradation of stagnant replication forks involves BRAC1- and BRAC2-deficient cells, thus conferring resistance to PARPi [101].

### 5.4. Next-Generation PARPi

Clinically approved PARP inhibitors have been shown to be effective against pancreatic, ovarian, prostate, breast, and other cancers, and although they provide significant benefits compared with standard chemotherapy, clinically approved PARP inhibitors have shown numerous adverse reactions [102]. Clinically approved PARP inhibitors are capable of inhibiting both PARP1 and PARP2, which renders the original function of PARP2 ineffective. A growing number of current-generation inhibitors are emerging. Studies have shown that a PARP inhibitor called AZD5305 has been developed that selectively captures only PARP1 but not PARP2 [103]. Selective inhibitors of PARP1 not only maintain anticancer efficacy but also reduce hematological toxicity. The majority of clinically approved PARP inhibitors target polyADP-ribosylase. Recent studies have shown that several inhibitors targeting mono-ADP-ribosylase have been developed sequentially, such as PARP10 inhibitor OUL35 [104], PARP14 inhibitor RBN012759 [105], PARP11 inhibitor ITK7, and PARP7 inhibitor RBN-2397 [106].

### 5.5. Advancements in PARPi Resistance Solutions

To address PARP resistance, numerous treatments for PARP resistance are also being promoted [107,108,109,110,111,112,113]. One approach is to remove the compactness of PARPi and HR defects, making PARPi independent of the BRCA1/2 mutation. The main mechanism is that PARPi promotes the accumulation of cytoplasmic DNA fragments caused by unresolved DNA lesions, thereby activating the corresponding DNA sensing pathway and stimulating the production of type I interferons to induce antitumor immunity independent of BRCAness. Immune checkpoint blocking further enhances these effects of PARPi [114]. The alternative is to replace PARPi with a PARG inhibitor to have a corresponding effect. Currently, there is also a research direction to address PARPi resistance by combining PARP inhibitors with alternative inhibitors. The combination of VEGF inhibitors and PARPi enhances treatment benefits in ovarian cancer. MEK inhibitors inhibit HR restoration and increase PARP expression, thereby enhancing the sensitivity of ovarian cancer patients to PARP therapy [115]. The combination of cell cycle checkpoint inhibitors and PARPi is also a good way to overcome PARPi resistance.

## 6. Conclusions and Future Prospects

In recent years, there has been a growing body of intensive research on ADP-ribosylation. Due to being the first member of the PARP family to be discovered, PARP1 has been most intensively studied and plays a huge role in the recruitment of DNA damage factors and DNA repair proteins, as well as in the crosstalk that occurs after ADP-ribosylation [112,116,117,118]. With the development of molecular tools, the functions of different PARP enzymes are also being characterized. Additionally, other types of ADP-ribosyl hydrolase have been found. Members of the cofactors involved in completing the ADP-ribosylation cycle have also been found in recent years. The increasing number of these factors demonstrate the crucial role of ADP-ribosylation in the DNA repair pathway.

Protein post-translational modification is the biochemical modification of specific amino acid residues on a target protein. Crosstalk in protein post-translational modification is a hot topic of current research. The regulation of ADP-ribosylation and its role in the DNA repair pathway is enhanced by the crosstalk between ADP-ribosylation and additional protein post-translational modifications. The crosstalk between PTMs has had a significant impact on various physiological activities and associated diseases. The crosstalk between ADP-ribosylation and various protein post-translational modification pathways is currently under in-depth investigation. With the discovery of new protein post-translational modifications, such as novel acylation modifications, ADP-ribosylation will be implicated in additional protein post-translational modification pathways.

PARPi holds significant potential as a therapeutic approach for the targeting of cancers associated with mutations in breast cancer susceptibility genes. However, certain limitations of PARPi in cancer therapy have been unveiled. Despite numerous proposed solutions to address these drawbacks, overcoming PARPi resistance remains a formidable challenge in the field of oncology. Combining PARPi with the inhibition of novel targets and more effective immunization strategies is essential for enhancing the efficacy of cancer treatment, necessitating further exploration and development of alternative strategies.

## Data Availability

No new unpublished data were included in this review.

## Abbreviations

ADPr	ADP-ribose
ARTs	ADP-ribosyltransferases
BAG3	bcl-2-associated athanogene 3
BAP1	BRCA1-associated protein 1
CAT	Catalytic domain
DDR	DNA-damage response
DSB	Double-strand break
GNAT	General control nonrepressible 5 (GCN5)-related N-acetyltransferase
HPF1	Histone PARylation factor 1
HR	Homologous recombination
MAR	Mono-ADP-ribosylation
NAD	Nicotinamide adenine
NAM	Nicotinamide
NAT10	N-acetyltransferase 10
NHEJ	Nonhomologous end junction
NUDT16	Nucleoside diphosphate-linked moiety X-type motif 16
PAR	Poly(ADP)-ribose
PARPi	PARP inhibitors
PFS	Progression-free survival
PNUTS	Phosphatase 1 nuclear0targeting subunit 1
PTMs	Post-translational modifications
SSB	SINGLE-strand break
STAT3	Signal transducer and activator of transcription 3
SUMO	Small ubiquitin-related modifier
TRIP12	Thyroid hormone receptor-interacting protein 12
TSG101	Tumor susceptibility gene 101 protein
